# Ranalexin-1G: A Promising Antimicrobial Peptide Targeting Virulence and Host–Pathogen Interactions in *Pseudomonas aeruginosa* In Vitro Models

**DOI:** 10.3390/antibiotics15070711

**Published:** 2026-07-22

**Authors:** Marina Acunzo, Carla Zannella, Rosa Giugliano, Laura Di Clemente, Carla Capasso, Annalisa Chianese, Maria Andriolo, Federica Donadio, Emanuela Esposito, Alessandra Monti, Nunzianna Doti, Teresa Maria Assunta Fasciana, Anna Giammanco, Massimiliano Galdiero, Anna De Filippis

**Affiliations:** 1 Department of Woman, Child and General and Specialized Surgery, University of Campania Luigi Vanvitelli, 80138 Naples, Italy; marina.acunzo@unicampania.it (M.A.); carla.zannella@unicampania.it (C.Z.); rosa.giugliano@unicampania.it (R.G.); laura.diclemente@unicampania.it (L.D.C.); carla.capasso@unicampania.it (C.C.); a.chianese@unilink.it (A.C.); massimiliano.galdiero@unicampania.it (M.G.); 2 UOC Virology and Microbiology, University Hospital “Luigi Vanvitelli”, 80138 Naples, Italy; 3 Department of Life Sciences, Health and Health Professions, Link Campus University, 00165 Rome, Italy; 4 Clinical Pathology Unit, Sant’ Elia Hospital, 93100 Caltanissetta, Italy; m.andriolo@asp.cl.it; 5 Institute of Applied Sciences and Intelligent Systems (ISASI), National Research Council (CNR), Naples Cryo-Electron Microscopy Laboratory (M6.1 EYE LAB), Via Pietro Castellino 111, 80131 Naples, Italy; federica.donadio@na.isasi.cnr.it (F.D.); emanuela.esposito@cnr.it (E.E.); 6 Institute of Biostructures and Bioimaging (IBB), National Research Council (CNR), 80131 Naples, Italy; alessandra.monti@ibb.cnr.it (A.M.); nunzianna.doti@cnr.it (N.D.); 7 Department of Health Promotion, Mother and Child Care, Internal Medicine and Medical Specialties, University of Palermo, 90127 Palermo, Italy; teresa.fasciana@unipa.it (T.M.A.F.); anna.giammanco@people.unipa.it (A.G.)

**Keywords:** antimicrobial peptides, *Pseudomonas aeruginosa*, cystic fibrosis isolates, antibacterial, host–pathogen interaction

## Abstract

Background: Lung infections represent a major cause of morbidity and mortality in patients with cystic fibrosis (CF) and are predominantly associated with chronic infection by *Pseudomonas aeruginosa*. The clinical management of CF lung disease is increasingly compromised by the emergence of multidrug-resistant strains, biofilm formation, and the expression of multiple virulence determinants. Antimicrobial peptides (AMPs), evolutionarily conserved effectors of innate immunity, have emerged as promising therapeutic candidates due to their ability to exert both bactericidal and anti-virulence activities. In this study, we investigated the antimicrobial and mechanistic effects of Ranalexin-1G, an AMP derived from the skin secretion of *Rana grylio*, against *P. aeruginosa*, for which its antibacterial activity has not previously been reported in the literature. Methods: Antibacterial activity was determined against the reference strain and three clinical isolates of *P. aeruginosa* by broth microdilution assays, time-kill kinetics and anti-biofilm assays, while peptide-mediated modulation of virulence was evaluated through transcriptional analysis of key virulence-associated genes by RT-PCR. The impact of Ranalexin-1G on host–pathogen interactions was further assessed using bacterial invasion assays in human bronchial epithelial cells (BEAS-2B). Results: Ranalexin-1G exerted a rapid bactericidal effect within 6 h at non-cytotoxic concentrations and displayed modest anti-biofilm effects, with greater efficacy in inhibiting biofilm formation. Mechanistically, peptide treatment resulted in a reduction in the expression of selected genes involved in biofilm formation and virulence, including those associated with alginate biosynthesis and type III secretion system-mediated cytotoxicity. Consistently, Ranalexin-1G markedly impaired bacterial invasion of epithelial cells, indicating interference with early host–pathogen interaction processes. Notably, the peptide displayed robust antimicrobial activity against multidrug-resistant *P. aeruginosa* clinical isolates from CF patients. Conclusions: Collectively, these findings suggest that Ranalexin-1G acts through a dual mechanism involving direct bactericidal activity and modulation of selected virulence pathways, supporting further investigation of its potential as an anti-virulence and host-directed approach for the treatment of chronic *P. aeruginosa* infections in cystic fibrosis.

## 1. Introduction

Cystic fibrosis (CF) is an inherited disorder transmitted through an autosomal recessive pattern, affecting approximately 89,000 individuals worldwide [1]. The condition originates from pathogenic variants in the CFTR (cystic fibrosis transmembrane conductance regulator) gene, located on human chromosome 7 (locus 7q.31) [2]. The *CFTR* gene encodes an ATP-binding cassette (ABC) anion channel expressed in the exocrine glands [3], mediating the transport of chloride and bicarbonate ions across the apical membranes of epithelial cells [4]. The loss or dysfunction of CFTR impairs chloride secretion and disrupts ion homeostasis, leading to increased epithelial sodium absorption and reduced airway surface liquid volume [4,5]. These alterations result in airway surface dehydration, impaired mucociliary clearance, and create a favorable environment for bacterial growth and biofilm formation [6]. Microbial infections induce a pronounced neutrophil-dominated inflammatory response, characterized by the release of proteolytic enzymes, including neutrophil elastase (NE), cathepsin G, metalloproteases and proteinase 3 [7]. While these proteases contribute to pathogen clearance, their activity also damages host tissues, exacerbating lung injury and perpetuating a self-sustaining cycle of inflammation and infection [8]. The resulting impairment of airway defenses and mucociliary clearance favors persistent respiratory infections, commonly caused by *Pseudomonas aeruginosa* (*P. aeruginosa*) [9]. *P. aeruginosa* has evolved multiple resistance mechanisms against antibiotics, including reduced antibiotic uptake through downregulation or modification of porins, enzymatic inactivation or modification of antimicrobial agents (e.g., production of β-lactamases, aminoglycoside-modifying enzymes), elevated mutation rates driven by hypermutator phenotypes, and adaptive behavioral changes such as biofilm formation and swarming motility regulation [6]. These combined strategies enable the pathogen to evade both antibiotic therapy and host immune defenses, thereby facilitating long-term persistence and chronic infection even in the presence of intensive antimicrobial treatment [10]. *P. aeruginosa* can establish both acute and chronic infections through several key mechanisms, including intrinsic antibiotic resistance [11], the ability to invade epithelial cells [12], toxin production, and biofilm formation.

Adhesion to host tissues is supported by multiple extracellular factors [13], including a self-produced extracellular polymeric substance (EPS) matrix that provides structural stability to the biofilm, and dispersal cells released from mature biofilms that contribute to the colonization of new surfaces. The genes involved in alginate biosynthesis are clustered in an operon encoding 12 proteins (AlgD-Alg8-Alg44-AlgK-AlgE-AlgG-AlgX-AlgL-AlgI-AlgJ-AlgF-AlgA), which coordinate polymer synthesis, modification, and export [14]. Alginate enhances bacterial surface adhesion, promotes biofilm formation, and protects cells from host immune defenses [13,15]. Following adhesion, *P. aeruginosa* employs type I–VI secretion systems (TSS) to deliver effector proteins that damage host cells and facilitate internalization [16,17,18]. Among these, the type III secretion system (T3SS) mediates the direct injection of effector proteins into host cells. Exoenzyme S (ExoS) is a well-characterized T3SS effector whose expression is induced upon contact with eukaryotic cells [19]. It is a bifunctional toxin that targets the host cytoskeleton and suppresses phagocytosis through its ADP-ribosyltransferase and GTPase-activating domains, increasing bacterial persistence [20]. In contrast, the type II secretion system (T2SS) is responsible for the extracellular release of virulence factors, including Exotoxin A (ExoA) and Elastase A (LasA). Exo A inhibits protein synthesis through ADP-ribosylation of elongation factor 2 (EF-2), which impairs mRNA translocation, disrupts protein homeostasis, and ultimately induces apoptosis [20]. LasA is a zinc-dependent metalloprotease [21], that contributes to tissue damage and facilitates bacterial invasion by degrading host proteins [18]. In response to bacterial infections, pulmonary cells overproduce mucus, which is primarily composed of mucins (MUCs) [22]. They are classified into secreted mucins (gel-forming mucins) and transmembrane mucins (membrane-bound mucins expressed on the apical surface of epithelial cells [23], and play crucial roles in lubricating epithelial tissues, cellular adhesion, and forming a barrier against pathogens, toxins, and mechanical stress [24]. Abnormal mucus production in the lungs leads to inflammation, hypoxia, and oxidative stress, exacerbating the clinical condition of cystic fibrosis patients [25].

Therefore, the combination of intrinsic and acquired antibiotic resistance mechanisms and strong virulence makes *P. aeruginosa* a challenging pathogen to treat in clinical settings [26], highlighting the need to explore and develop new broad-spectrum antimicrobial agents [11].

Antimicrobial peptides (AMPs) are natural oligopeptides discovered in a wide range of organisms, including bacteria, insects, and amphibians [27,28]. They exhibit common physicochemical properties, such as a small size (ranging from 10 to 50 amino acid residues) and a predominantly hydrophobic nature [29]. AMPs can be classified based on their net charge into cationic antimicrobial peptides (CAMPs) and anionic antimicrobial peptides (AAMPs) [30]. Structurally, they are grouped into four main classes: α-helical, β-sheet, extended, and cyclic (looped) peptides [31]. Antimicrobial peptides are known for their ability to bind and compromise microbial cells by pore formation via the barrel stave mechanism or carpet formation [32]. However, the antimicrobial activity of several naturally occurring AMPs may be reduced under physiological conditions, including high ionic strength and serum- or mucus-rich environments, which are particularly relevant to the cystic fibrosis lung microenvironment [33,34]. AMPs isolated from frog skin secretions are an extremely heterogeneous class of peptides, which share three common characteristics that make them attractive for antimicrobial use: (i) the presence of basic amino acids that confer a positive charge, (ii) the presence of at least 50% of hydrophobic amino acids, and (iii) the ability to form amphipathic alpha-helix and/or beta-sheet structures following their interaction with the phospholipid membrane of the target cell [35]. Among the best-known families of AMPs are the Temporins, isolated from the skin secretions of the European common frog *Rana temporaria*. These short, hydrophobic pep-tides exhibit potent activity primarily against Gram-positive pathogens, with minimum inhibitory concentrations (MCs) typically ranging between 2.5 and 20 µM [36]. In addition to temporins, the skin of ranid frogs produces the Ranalexins, another crucial family of AMPs evolutionary linked to the structural framework of polymyxins [37]. Ranalexins are small, cationic molecules characterized by a conserved C-terminal heptapeptide loop formed by a Cys14–Cys20 disulfide bridge. For the archetype of this family, the Ranalexin isolated from the American bullfrog *Rana catesbeiana*, the biophysical evaluations, including circular dichroism and NMR spectroscopy, have revealed a fascinating structural trait. Specifically, both the reduced (linear) and oxidized (cyclic) forms of the peptide adopt highly identical amphipathic α-helical conformations in membrane-mimetic environments (like DPC or SDS micelles). Crucially, both forms display comparable antimicrobial activity, definitively proving that the covalent disulfide constraint is dispensable for both mem-brane-pore formation and overall biological function [38]. This structural flexibility represents a major advantage for affordable, large-scale synthetic manufacturing, as it bypasses the need for complex in vitro oxidative folding. Between Ranalexins, the Ranalexin-1G, isolated from the pig frog *Rana grylio* and central to this study, represents a highly homologous 20-residue analogue that perfectly preserves the Cys14–Cys20 motif [38]. Unlike the Gram-positive-skewed profile of early ranalexins, Ranalexin-1G exhibits broad-spectrum antimicrobial activity: growth-inhibitory against Gram-positive bacteria (e.g., *Staphylococcus aureus*), Gram-negative bacteria (e.g., *Escherichia coli*), and fungi/yeasts (e.g., *Candida albicans*). Reported MIC values are in the low micromolar range (e.g., ~9 µM for *E. coli*, ~18 µM for *S. aureus*, ~70 µM for *C. albicans*). These robust, low-micromolar thresholds, highlight Ranalexin-1G as an exceptionally versatile scaffold for the engineering of next-generation peptide antibiotics against pan-resistant superbugs. In the present study, we investigated its efficacy against clinical isolates of *P. aeruginosa* obtained from CF patients along with the standard strain (*P. aeruginosa* ATCC 13388). In addition, we evaluated its anti-virulence effects, including its impact on key pathogenic mechanisms, and assessed its interactions with airway epithelial cells in relevant in vitro models, aiming to further characterize the therapeutic potential of this antimicrobial peptide.

## 2. Results

### 2.1. Ranalexin-1G Synthesis and Characterization

**Figure 1:**
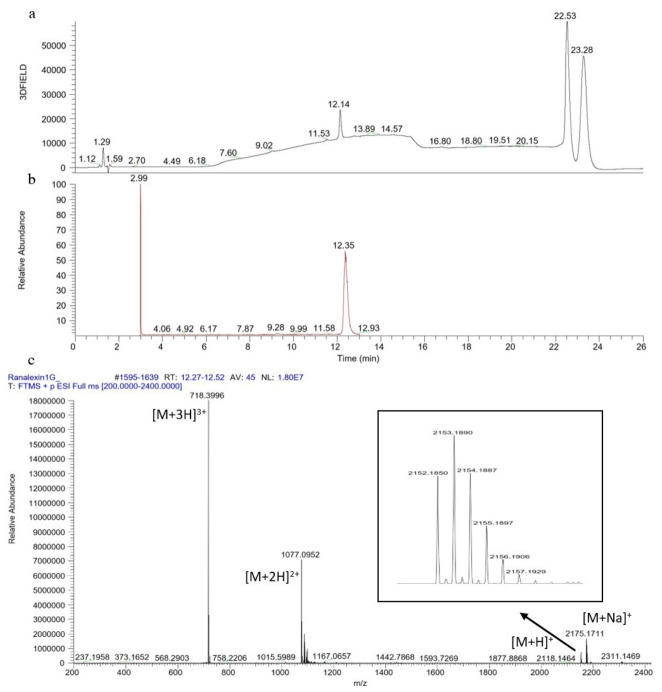
LC-MS characterization of Ranalexin-1G. (**a**) Representative RP-HPLC-DAD chromatogram showing the purified peptide eluting at a retention time (Rt) of 9.8 min. (**b**) Total ion chromatogram (TIC) from the LC-MS analysis. (**c**) High-resolution MS spectrum displaying the characteristic ion signals: *m*/*z* 2175.167 [M+Na]^+^, *m*/*z* 2152.472 [M+H]^+^, *m*/*z* 1077.095 [M+2H]^2+^, and *m*/*z* 718.399 [M+3H]^3+^. These experimental values are consistent with the calculated monoisotopic molecular weight (MW) of 2151.160 Da. The inset shows the isotopic profile of the [M+H]^+^ peak at *m*/*z* 2152.185. The reported *m*/*z* values correspond to the monoisotopic or most abundant peaks within each isotopic cluster.

Ranalexin-1G (primary sequence: FLGGLMKIIPAAFCAVTKKC), N-terminally acetylated and C-terminally amidated, was synthesized using Fmoc solid-phase peptide synthesis (SPPS) on a Rink Amide resin. Following synthesis, the crude peptide was purified to homogeneity via reversed-phase high-performance liquid chromatography (RP-HPLC). The identity and purity of the final product were confirmed by liquid chromatography–mass spectrometry (LC-MS) analysis (Figure 1), which demonstrated a high degree of purity (>95%), rendering the peptide suitable for all subsequent experimental applications.

With the aim of investigating the activity of the peptide strictly in its reduced state, and given the presence of two cysteine residues, time-course analyses (0, 2, and 24 h) were performed at pH 7.4 in both phosphate and bicarbonate buffers to evaluate its stability and oxidation state. Under all tested conditions, the peptide consistently remained in its reduced form, even in the absence of reducing agents (Supplementary Figure S1). Consequently, all subsequent experiments were carried out without reducing agents.

### 2.2. Cytotoxic Profile of Ranalexin-1G

**Figure 2:**
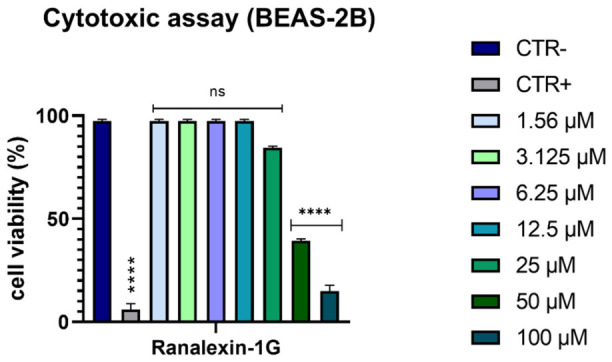
Assessment of the cytotoxicity profile of Ranalexin-1G. Cytotoxicity was assessed in the BEAS-2B cell line using an MTT assay after 24 h of peptide treatment. The data represent the mean ± SD of three independent experiments. CTR−: untreated cells; CTR+: treated cells with 100% Dimethyl Sulfoxide (DMSO). Statistical analysis was performed using Dunnett’s multiple comparisons test: **** *p*-value < 0.0001; ns *p*-value > 0.05.

**Figure 3:**
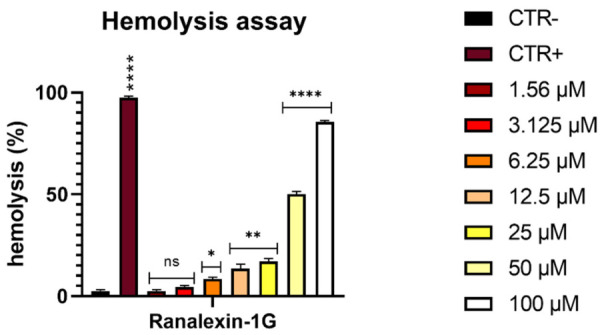
Hemolytic activity of human erythrocytes after Ranalexin-1G treatment. The data represent the mean ± SD of three independent experiments. CTR−: untreated cells; CTR+: 1% Triton X-100. Statistical analysis was performed using Dunnett’s multiple comparisons test: **** *p*-value < 0.0001; ** *p*-value = 0.0028; * *p*-value = 0.0358; ns *p*-value > 0.05.

The cytotoxicity of Ranalexin-1G was evaluated in human lung epithelial cells (BEAS-2B) cells exposed to concentrations ranging from 1.56 to 100 µM for 24 hours using the 3-(4,5-dimethylthiazol-2-yl)-2,5-diphenyltetrazolium bromide (MTT) assay, while its hemolytic activity was assessed in human erythrocytes. The data presented in Figure 2 show that the peptide exhibits low toxicity, with cell viability values around 85% even at a concentration of 25 µM (CC_50_ = 40.5 µM). Hemolysis assay (Figure 3) revealed limited hemolytic activity at lower concentrations, reaching 15% and 21% erythrocyte lysis at 12.5 µM and 25 µM of Ranalexin-1G, respectively (HC_50_ = 50 µM).

### 2.3. Antibacterial Activity

**Figure 4:**
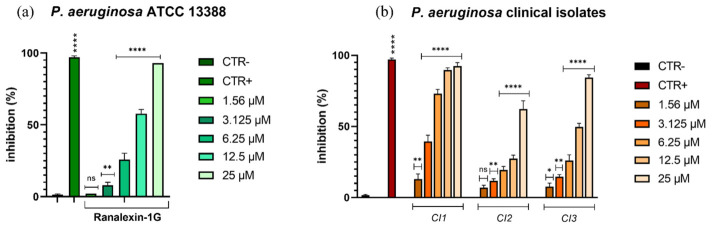
Growth inhibition rate (%) of *P. aeruginosa* strains after 20 h treatment with Ranalexin-1G at a concentration range of 1.56 ÷ 25 μM. (**a**) *P. aeruginosa* ATCC 13388 (**b**) *P. aeruginosa* clinical isolates. CTR−: untreated bacteria; CTR+: bacteria challenged with Meropenem (25 μM). Data are expressed as mean ± SD of three independent biological replicates. Statistical significance was determined using one-way ANOVA, comparing each treated group to untreated bacteria, as follows: **** *p*-value < 0.0001; ** *p*-value = 0.0024; * *p*-value = 0.0030; ns *p*-value > 0.05.

**Figure 5:**
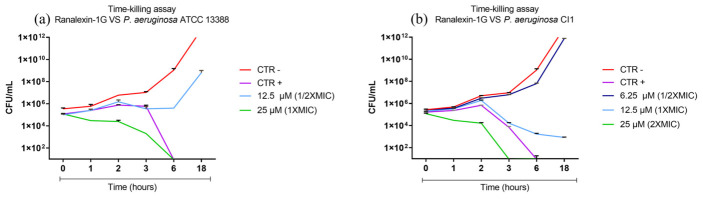
Time-kill kinetics of Ranalexin-1G against *P. aeruginosa* ATCC 13388 (**a**) and clinical isolate 1 (**b**). Untreated bacteria and Meropenem-treated (25 µM) bacteria served as CTR− and CTR+, respectively.

The antibacterial efficacy of Ranalexin-1G was evaluated within its non-cytotoxic range (1.56–25 µM) against *P. aeruginosa* ATCC 13388 and three clinical isolates using the broth microdilution method, in accordance with EUCAST guidelines. Meropenem, a broad-spectrum carbapenem tested at ~25 μM, served as a positive control (CTR+). As shown in Figure 4a, Ranalexin-1G exhibited antibacterial activity against *P. aeruginosa* ATCC 13388, with a minimum inhibitory concentration (MIC) value of 25 µM (Table 1). In addition, the peptide showed MIC values in the range of 12.5–25 µM against clinical isolates (Figure 4b and Table 1). Minimum bactericidal concentration (MBC) values were determined by spotting the broth from MIC, ½ × MIC, and 2 × MIC assays onto agar plates. The obtained results showed that Ranalexin-1G exhibits a bactericidal effect at 25 µM against *P. aeruginosa* ATCC 13388 and clinical isolate 1, whereas it displays a bacteriostatic effect against clinical isolates 2 and 3. Finally, a time-kill kinetics analysis was performed against *P. aeruginosa* ATCC 13388 and clinical isolate 1 at 2 × MIC, 1 × MIC, ½ × MIC to evaluate the rate of Ranalexin-1G action. Results demonstrated that Ranalexin-1G completely eradicated bacterial cells of the *P. aeruginosa* reference strain within 6 h at its MIC (25 µM), whereas no bactericidal activity was observed at ½ × MIC (Figure 5a). In contrast, for clinical isolate 1, the peptide achieved complete bacterial eradication after 3 h at 2 × MIC (25 µM), whereas no bactericidal activity was observed at 1 × MIC (12.5 µM) (Figure 5b). This finding is consistent with the MBC results, which demonstrated that the peptide exhibited a bacteriostatic effect at 12.5 µM.

### 2.4. Ranalexin-1G Impact on the Genes Involved in Alginate Biosynthesis

**Figure 6:**
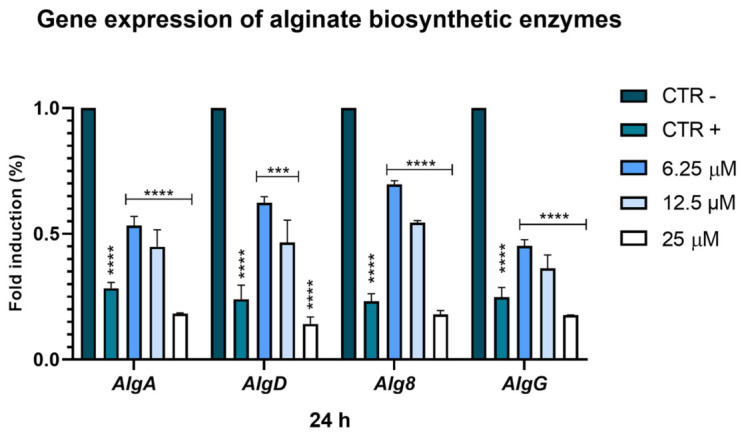
Molecular assay. Real-time PCR was performed to evaluate the effect of Ranalexin-1G on the expression of *P. aeruginosa* genes involved in alginate biosynthesis, specifically *algA*, *algD*, *alg8*, and *algG*. CTR− refers to untreated bacteria. CTR+: bacteria exposed to Meropenem (25 µM). Data represent mean ± standard deviation (SD) of three independent experiments. Statistical significance was determined, comparing peptide-treated samples to CTR−, as follows: **** *p*-value < 0.0001; *** *p*-value < 0.0006.

To better understand the effect of Ranalexin-1G on the virulence factors of *P. aeruginosa*, we analyzed the expression of genes involved in alginate biosynthesis, an exopolysaccharide that plays a key role in biofilm formation, immune evasion, and bacterial adhesion. The alginate operon in *P. aeruginosa* consists of 12 genes, and we assessed *algA*, *algD*, *algG*, and *alg8*, using Real-Time PCR [39,40]. The results showed that Ranalexin-1G downregulated gene expression, with the most significant effect observed for *algD* in bacterial cells treated with 25 µM of Ranalexin-1G (Figure 6).

### 2.5. Effect of Ranalexin-1G on P. aeruginosa Biofilm

**Figure 7:**
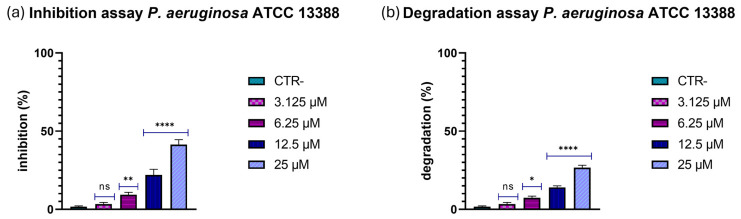
Evaluation of anti-biofilm activity of Ranalexin-1G against *P. aeruginosa*. (**a**) Effect of Ranalexin-1G during biofilm formation (**b**) activity against preformed biofilm. Data represent mean ± standard deviation (SD) of three independent experiments. Statistical analysis was performed by One-way ANOVA followed by Dunnett’s multiple comparisons test. Significances are referred to the untreated sample (CTR−). (**a**) **** *p* < 0.0001; ** *p* = 0.0062; ns: non-significant; (**b**) **** *p* < 0.0001; * *p* = 0.0389; ns *p*-value > 0.05.

Biofilm formation represents one of the major virulence determinants associated with *P. aeruginosa*, contributing significantly to the persistence of chronic respiratory infections and disease progression in patients with cystic fibrosis. The activity of the peptide was evaluated both during the biofilm formation phase, using the inhibition assay, and against preformed mature biofilms, using the degradation assay. In the present study, biofilms were exposed to peptide concentrations ranging from 25 to 3.125 µM, and biomass was quantified by crystal violet (CV) staining method. Ranalexin-1G showed a moderate antibiofilm activity in both experimental approaches, with the most pronounced effect observed in the biofilm inhibition assay. At the highest tested concentration (25 µM), the peptides reduced biofilm formation by 41% (Figure 7a). Under the same conditions, the biofilm degradation assay (Figure 7b) revealed a reduction in biofilm biomass of 26%.

### 2.6. Bacterial Internalization Assay

**Figure 8:**
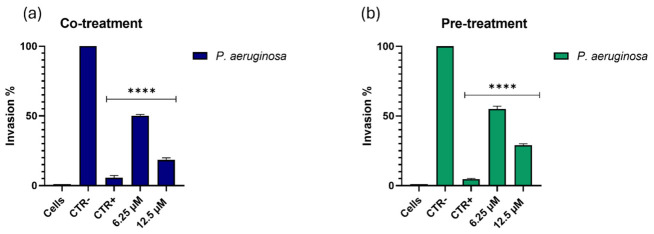
Invasion assay of *P. aeruginosa* in BEAS-2B cells. (**a**) Co-treatment: Cells were treated with two different concentrations of Ranalexin-1G and infected with *P. aeruginosa* at the same time (**b**) Pre-treatment: Cells were treated with two different concentrations of Ranalexin-1G and then infected with *P. aeruginosa*. Cells: Untreated cells; CTR−: cells infected with *P. aeruginosa* (MOI = 25); CTR+: cells exposed to Meropenem (25 μM). Data are reported as % of *P. aeruginosa* cell invasion and represent mean values + standard errors of the means of triplicate experiments. Statistical significance was determined, comparing peptide-treated samples to untreated cells, as follows: **** *p*-value < 0.0001.

BEAS-2B cells were infected with *P. aeruginosa* ATCC 13388 at a multiplicity of infection (MOI) of 25 and treated with Ranalexin-1G at concentrations of 12.5 and 6.25 µM. Two experimental approaches were employed (co-treatment and pre-treatment assays) to investigate the potential effect of the peptide on bacterial internalization into epithelial cells. In co-treatment conditions, epithelial cells were exposed to Ranalexin-1G simultaneously with the bacterial infection. Under these conditions, a marked reduction in the number of intracellular bacteria was observed. Specifically, the bacterial invasion rate decreased to approximately 20% at 12.5 µM and to 50% at 6.25 µM compared with the untreated infected control (Figure 8a). These results indicate a strong inhibitory effect of the peptide on *P. aeruginosa* internalization when present during bacterial infection. In the pre-treatment assay, BEAS-2B cells were pre-incubated with Ranalexin-1G for 24 h prior to infection with *P. aeruginosa*. In this experimental setting, the reduction in bacterial invasion was slightly less pronounced, with invasion rates of approximately 30% and 55% observed at 12.5 µM and 6.25 µM, respectively (Figure 8b). Although the inhibitory effect remained evident, these values suggest that the peptide exerts a stronger activity when directly present during the interaction between bacteria and host cells.

### 2.7. Modulation of Virulence Gene Expression and Host Mucin Response

**Figure 9:**
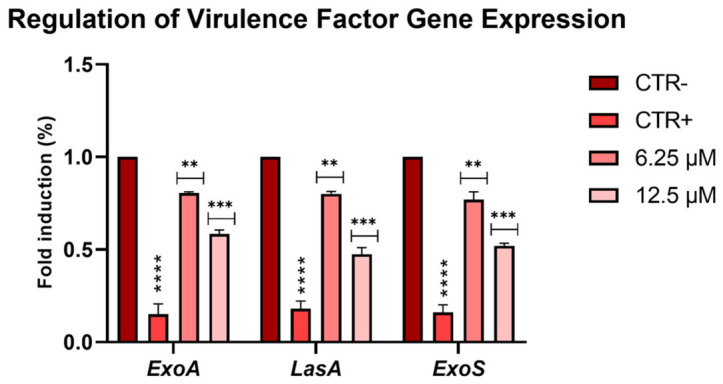
Molecular assay. Real-time PCR was performed to evaluate the effect of Ranalexin-1G on genes associated with *P. aeruginosa* invasion and host cell damage, specifically *exoA*, *lasA* and *exoS* genes. CTR− refers to untreated bacteria. CTR+: bacteria exposed to Meropenem (25 µM). Data represent mean ± standard deviation (SD) of three independent experiments. Statistical significance was determined, comparing peptide-treated samples to CTR−, as follows: **** *p*-value < 0.0001; *** *p*-value < 0.0006; ** *p*-value = 0.0025.

*P. aeruginosa* secretes numerous virulence factors that contribute to host cell damage and promote pathogenesis during infection. Particularly relevant are *exoS*, *exoA*, and *lasA*, which are directly involved in host cell invasion and tissue injury. As shown in Figure 9, real-time PCR analysis confirmed that Ranalexin-1G can modulate the expression of genes encoding these proteins in dose dependent manner. At a concentration of 6.25 µM, the peptide already achieves a statistically significant reduction in mRNA transcript levels compared to the untreated control. Increasing the dosage to 12.5 µM further suppresses the expression of genes, reducing the induction of these virulence factors to approximately 50% of the untreated control. Considering the impact of bacterial virulence factors on epithelial cell responses, we investigated whether Ranalexin-1G could also regulate airway mucin gene expression in infected cells. Mucin gene expression is highly tissue-specific [41]. Therefore, in this study, we evaluated the regulation of three airway mucins (*MUC1*, *MUC16*, and *MUC5AC*). MUC1 and MUC16 are the two major membrane mucins expressed on the apical membranes of epithelial cells in the respiratory system [42], whereas MUC5AC is the predominant component of the mucus gel in normal airways and contributes to the barrier function of airway mucus [43]. These mucins were selected as representative markers of epithelial barrier response and infection-induced mucosal activation, which are typically upregulated during bacterial challenge as part of the host defensive response.

**Figure 10:**
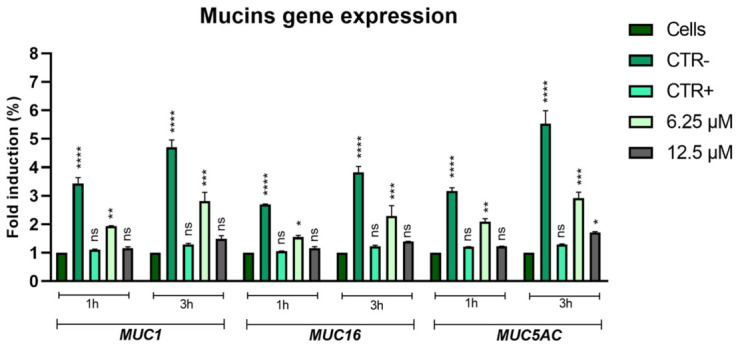
Molecular assay. Real-time PCR was performed to evaluate the effect of Ranalexin-1G on genes associated with BEAS-2B cells’ mucus secretion. The expression levels of *MUC1*, *MUC16*, and *MUC5AC* genes were analyzed. Cells: Untreated cells; CTR−: cells infected with *P. aeruginosa* (MOI = 25); CTR+: cells exposed to Meropenem (25 µM). Data represent mean ± standard deviation (SD) of three independent experiments. Statistical significance was determined, comparing peptide-treated samples to cells, as follows: **** *p*-value < 0.0001; *** *p*-value < 0.0006; ** *p*-value = 0.0025; * *p*-value = 0.040; ns *p*-value > 0.05.

BEAS-2B cells showed basal expression of these mucins, which was further upregulated following bacterial infection. Total RNA was extracted at different time points (1 and 3 h) after treatment, and we observed that Ranalexin-1G significantly decreased mucin transcript levels, with the maximal reduction observed after 3 h at a concentration of 12.5 µM (Figure 10).

### 2.8. Ranalexin-1G Induces Morphological Changes in P. aeruginosa

**Figure 11:**
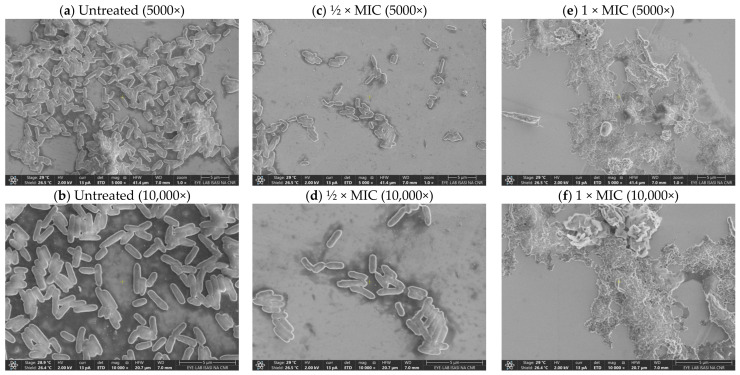
SEM analysis of *P. aeruginosa* cells treated with different concentration of Ranalexin-1G. The images were acquired at 5000× and 10,000× magnifications. (**a**,**b**) Untreated bacteria, (**c**,**d**) bacteria treated with ½ × MIC, (**e**,**f**) bacteria treated with MIC.

The Ranalexin-1G antibacterial potential against *P. aeruginosa* reference strain was corroborated by Scanning electron microscopy (SEM) analysis. Untreated bacterial cells exhibited the typical rod-shaped morphology with a smooth and intact surface, consistent with normal cellular integrity (Figure 11a,b). In contrast, cells exposed to the peptide displayed evident morphological alterations and structural damage. In particular, treatment with the higher peptide concentration (1 × MIC) resulted in pronounced cellular damage, characterized by severe deformation of the bacterial envelope, collapse of the cell structure, and clear loss of membrane integrity. These alterations were accompanied by the presence of abundant cellular debris and disrupted bacterial cells (Figure 11e,f). At the lower concentration (½ × MIC), morphological alterations were still detectable; however, the damage appeared less extensive compared with that observed at 1 × MIC. Nevertheless, a noticeable reduction in the number of intact bacterial cells was observed, suggesting a decrease in bacterial load, together with subtle morphological changes, such as partial aggregation and surface irregularities. (Figure 11c,d). Overall, SEM observations confirmed that Ranalexin-1G exerts a strong antibacterial effect against *P. aeruginosa*, inducing significant structural damage to bacterial cells and markedly reducing the number of viable bacteria.

### 2.9. Evaluation of Bacterial Membrane Integrity

**Figure 12:**
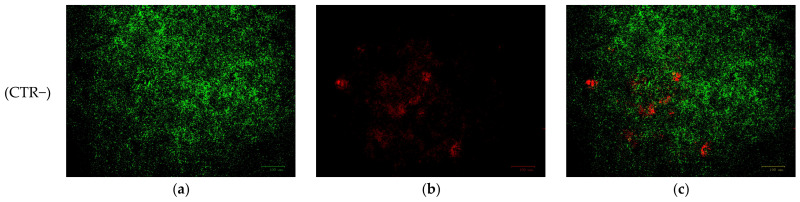

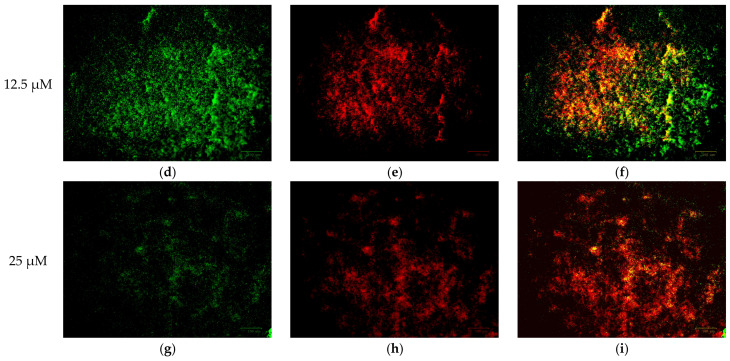
Images of *P. aeruginosa* cells stained with LIVE/DEAD kit, acquired via fluorescence microscopy. (**a**–**c**) untreated cells (CTR−); (**d**–**f**) bacteria treated with 12.5 μM of Ranalexin-1G; (**g**–**i**) bacteria treated with 25 μM of Ranalexin-1G.

To further validate the membrane damage observed by SEM, a LIVE/DEAD fluorescence assay was performed on *P. aeruginosa* cells following treatment with Ranalexin-1G (Figure 12). Untreated cells (CTR−) exhibited predominantly green fluorescence (SYTO 9), indicating a viable bacterial population with intact membranes, while propidium iodide (PI) staining was almost undetectable (Figure 12a–c). In contrast, treatment with Ranalexin-1G resulted in a concentration-dependent increase in red fluorescence, consistent with progressive membrane permeabilization. At 12.5 μM, a substantial proportion of PI-positive cells was already evident, indicating loss of membrane integrity in a significant fraction of the bacterial population (Figure 12d–f). Further increasing the peptide concentration to 25 μM led to a marked decrease in the overall cell number and a concomitant increase in red fluorescence, with PI-positive cells becoming predominant, indicating extensive membrane disruption (Figure 12g–i). The merged images revealed a progressive shift from viable (green) to membrane-compromised (red) bacteria with increasing peptide concentration. These findings are consistent with the ultrastructural alterations observed by SEM and further support that the antibacterial activity of Ranalexin-1G is mediated through disruption of the bacterial membrane.

### 2.10. Antimicrobial Activity in the Presence of Salts

**Figure 13:**
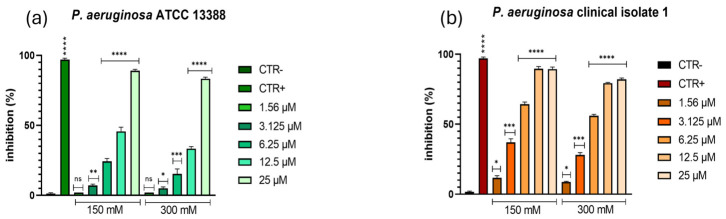

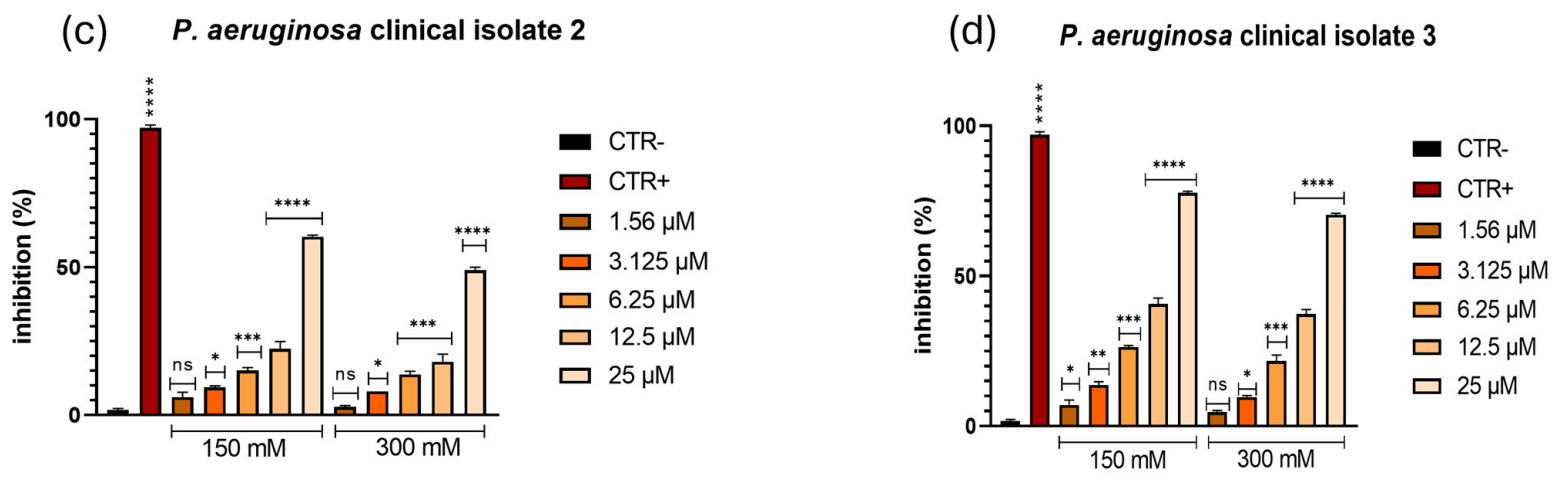
Effect of 150 and 300 mM of NaCl on the antibacterial activity of Ranalexin-1G against (**a**) *P. aeruginosa* ATCC 13388, (**b**) *P. aeruginosa* clinical isolate 1, (**c**) *P. aeruginosa* clinical isolate 2, (**d**) *P. aeruginosa* clinical isolate 3. CTR−: untreated bacteria; CTR+: bacteria challenged with Meropenem (25 μM). Statistical significance was determined, comparing peptide-treated samples to CTR−, as follows: **** *p*-value < 0.0001; *** *p*-value < 0.0002; ** *p*-value = 0.0012; * *p*-value = 0.0030; ns *p*-value > 0.05.

The impact of cationic salts on the growth inhibitory activity of Ranalexin-1G was evaluated using broth microdilution assays performed in MH broth supplemented with 150 or 300 mM NaCl. As shown in Figure 13, the presence of 150 mM NaCl did not affect the antimicrobial activity of the peptide, as the inhibitory effect remained comparable to that observed under salt-free conditions. In contrast, exposure to 300 mM NaCl resulted in a moderate decrease in the percentage of inhibition compared with the assay performed in the absence of salt. 

## 3. Discussion

The rising global threat posed by multidrug-resistant (MDR) *P. aeruginosa* underscores the urgent need for novel therapeutic agents capable not only of killing bacteria but also of attenuating virulence and mitigating host tissue damage [44]. In this context, the identification of broad-spectrum antimicrobial molecules is essential to expand current therapeutic options. In the present study, we investigated the ability of Ranalexin-1G to counteract *P. aeruginosa* infection, as well as its potential to modulate bacterial virulence and host–pathogen interactions.

Analytical characterization evidenced that the peptide is stable in its reduced form; under these conditions, it exhibited a low cytotoxic profile in human BEAS-2B bronchial epithelial cells (Figure 2), alongside modest hemolytic effect (Figure 3). Concurrently, it demonstrated significant antibacterial activity against both a reference strain and clinical isolates from CF patients (Figure 4), particularly within the first six hours of infection (Figure 5a,b). Selectivity index analysis revealed SI values of ~3.7 (CC_50_/IC_50_) for the reference strain, indicating a modest yet measurable selectivity toward bacterial cells over mammalian cells. Notably, the peptide remained active at both 150 and 300 mM NaCl, despite a moderate reduction in antibacterial activity at 300 mM, indicating good tolerance to saline conditions (Figure 13). Furthermore, we evaluated the anti-biofilm activity of Ranalexin-1G, considering that biofilm formation represents a key virulence determinant of *P. aeruginosa*, particularly in the context of chronic infections associated with cystic fibrosis. Our findings demonstrated that, at the highest concentration tested (25 μM), the peptide significantly impaired biofilm development, reducing biofilm formation by 41% (Figure 7a). Moreover, Ranalexin-1G exhibited the ability to affect pre-established mature biofilms, inducing a 26% reduction in biofilm biomass (Figure 7b). Ranalexin-1G activity was further supported by SEM analysis, which revealed pronounced morphological alterations and loss of membrane integrity following peptide treatment (Figure 11). At a concentration of 1 × MIC, bacterial cells displayed severe structural damage, including disrupted envelopes, indicating compromised membrane integrity. These observations were corroborated by LIVE/DEAD staining, revealing a concentration-dependent effect on membrane integrity. Bacteria treated with 25 μM showed extensive membrane damage, while those exposed to 12.5 μM displayed a more limited alteration.

The cationic nature of Ranalexin-1G likely facilitates electrostatic interactions with anionic lipids in the bacterial membrane, such as phosphatidylglycerol, cardiolipin, and lipopolysaccharides, thereby destabilizing membrane structure, permeability, and fluidity [45]. In contrast, mammalian cell membranes are predominantly composed of zwitterionic phospholipids, including phosphatidylcholine and sphingomyelin, and are enriched in cholesterol, which reduces overall negative surface charge and increases membrane rigidity [46,47].

Consistent with this mechanism, numerous studies have highlighted the antibacterial potential of frog-derived AMPs that act primarily through membrane disruption [48,49,50]. For instance, Maolin Tian et al. (2021) reported that brevinin-2MP, a peptide derived from the skin of *Microhyla pulchra*, induces significant bacterial membrane damage [51].

Beyond its direct antimicrobial activity, Ranalexin-1G markedly interfered with the virulence machinery of *P. aeruginosa*, leading to a significant reduction in bacterial internalization into lung epithelial cells. This finding is particularly relevant, as intracellular persistence represents an important survival strategy for *P. aeruginosa*. Alginate production is a hallmark of chronic infection, especially in CF, as it forms a protective matrix that shields bacteria from host immune responses and antibiotic treatment. Molecular analyses revealed significant downregulation of the alginate biosynthesis operon, particularly *algD*, which encodes a key checkpoint enzyme in the pathway. Reduced expression of *algA* (phosphomannose isomerase), *algD* (GDP-mannose dehydrogenase), *alg8* (alginate polymerase), and *algG* (C5-mannuronan epimerase) suggests that Ranalexin-1G impairs the ability of *P. aeruginosa* to establish persistent biofilms.

Moreover, the suppression of *exoS* and *exoA* genes is highly relevant to the clinical progression of *P. aeruginosa* infections. *exoS* encodes a major effector of the Type III secretion system (T3SS), while *exoA* inhibits host protein synthesis. Their downregulation, even at non-inhibitory concentrations, suggests a reduced bacterial capacity to induce epithelial cell death. *lasA* is a gene that encodes a protease responsible for degrading host connective tissue. The reduction in *lasA* expression suggests that Ranalexin-1G may mitigate tissue damage and slow the pathogen’s spread in respiratory environments. Collectively, the suppression of *exoS*, *exoA*, and *lasA*—genes associated with T3SS-mediated toxicity and elastase activity—indicates that Ranalexin-1G reduces the virulence arsenal used by *P. aeruginosa* to damage host tissues and evade immune clearance.

The interaction between *P. aeruginosa* and the respiratory epithelium is a decisive factor in the severity of infection. Using bacterial internalization assays, we evaluated peptide activity at different stages of infection through co-treatment and pre-treatment approaches. At a concentration of 12.5 μM, Ranalexin-1G reduced bacterial invasion by more than 80% during co-treatment. Previous studies suggest that AMPs may exert their effects not only through membrane disruption but also via internalization into prokaryotic cells, where they can interfere with secretion systems and modulate the release of virulence factors [52,53]. Additionally, AMPs have been shown to enter eukaryotic cells—despite differences in membrane composition—either via G protein-coupled receptors or through direct translocation, subsequently modulating host immune responses [54]. The greater efficacy observed during co-treatment compared to pre-treatment suggests that Ranalexin-1G may act during the early stages of bacterial interaction with host cells or may neutralize surface-associated virulence factors before they trigger endocytosis in BEAS-2B cells. However, it should be noted that, under co-treatment conditions, a minor contribution of extracellular effects prior to bacterial internalization cannot be completely excluded, even though sub-MIC peptide concentrations were used. Finally, Ranalexin-1G significantly modulated host mucin expression. While mucins such as MUC1, MUC16, and MUC5AC are essential for epithelial barrier function, their pathological overproduction leads to airway obstruction and creates a favorable niche for bacterial colonization [42,43]. The observed downregulation of these mucins three hours post-infection indicates that Ranalexin-1G may modulate infection-associated host responses, an effect that could be partially related to its impact on bacterial burden. This effect is especially relevant in CF, where excessive mucus production promotes chronic colonization and biofilm formation. Importantly, all gene expression analyses were deliberately performed using sub-inhibitory concentrations of Ranalexin-1G, selected to minimize the impact of bacterial growth inhibition or loss of viability on transcriptional responses. Since these concentrations produced only limited antibacterial effects, the observed downregulation of virulence-associated genes is less likely to reflect a nonspecific consequence of impaired bacterial fitness and instead supports the hypothesis that Ranalexin-1G interferes with virulence-related pathways. Nevertheless, further investigations will be required to elucidate the molecular mechanisms underlying this effect and to determine whether the peptide directly modulates virulence regulatory networks. Taken together, these findings suggest that Ranalexin-1G exerts bactericidal activity and may influence host–pathogen interactions, supporting its potential as a promising candidate for further preclinical evaluation as an anti-virulence and host-modulating strategy against *P. aeruginosa* infections. Furthermore, the pronounced activity of the peptide in its reduced form, consistent with previous reports [38], suggests that the two cysteine residues represent primary targets for modification to improve peptide stability and/or enhance antimicrobial and host-directed activity.

## 4. Materials and Methods

### 4.1. Peptide Synthesis and Characterization

All commercially available solvents and reagents for peptide synthesis and characterization were used in their original form without further purification. Solvents, including acetonitrile (CH_3_CN), dimethylformamide (DMF), dichloromethane (DCM), tetrahydrofuran (THF), and methanol (CH_3_OH), as well as protected amino acids and coupling agents such as: HATU (1-[Bis(dimethylamino)methylene]-1H-1,2,3-triazole[4,5-b]pyridine-3-oxide hexafluorophosphate), HOBt (1-Hydroxybenzotriazole hydrate), Oxyma (ethyl 2-cyano-2-(hydroxyimino)acetate), and diisopropylcarbodiimide (DIC), used for peptide synthesis were obtained from Deltek s.r.l. (Naples, Italy). Other products, such as N,N-di-isopropylethylamine, piperidine, piperazine, morpholine, 2,4,6-trimethylpyridine (sym-collidine), acetic anhydride (Ac_2_O), and trifluoroacetic acid (TFA), dithiothreitol (DTT) were purchased from Sigma-Aldrich (Merck Life Science S.r.l., Milan, Italy). The N-terminally acetylated and C-terminally amidated Ranalexin-1G peptide (CH_3_CO-FLGGLMKIIPAAFCAVTKKC-CONH_2_) was synthesized on Rink Amide resin using an optimized Fmoc solid-phase peptide synthesis protocol therefore yielding the expected C-terminal amide [55]. Cleavage of the peptide from the solid support was performed by treating the resin with 1 mL of a TFA/TIS/H_2_O mixture (90:5:5, *v*/*v*/*v*) for 3 h at room temperature. Due to the presence of cysteine residues, an additional aliquot of TIS (50 µL) was added to the cleavage mixture. The crude peptide was then precipitated with cold diethyl ether, dissolved in a 50:50 (*v*/*v*) mixture of H_2_O and CH_3_CN, and lyophilized. The crude material was treated with DTT (5 equivalents per cysteine) for 1 h at room temperature to reduce any disulfides and prevent intermolecular dimerization prior to purification. Peptide purification was carried out by high-performance liquid chromatography (HPLC) using a WATERS 2545 system (Waters, Milan, Italy), equipped with a WATERS 2489 UV/Vis detector. Separation was achieved using a C18 Jupiter column (5 μm, 300 Å, 250 × 21.2 mm) with a linear gradient of CH_3_CN-0.1% TFA in H_2_O-0.1% TFA from 20% to 80% over 15 min at a flow rate of 15 mL/min. Peptide identification and characterization were performed using an Orbitrap high-resolution mass spectrometer (Q-Exactive Plus) (ThermoFisher Scientific, Ferentino, Italy), with a C18 Aeris column (3.6 μm, 100 × 2.1 mm), employing a linear gradient of CH_3_CN/0.05% TFA in H_2_O/0.05% TFA from 1% to 85% over 8 min at a flow rate of 0.2 mL/min. The final purified peptide was obtained with a yield of approximately 80% and a purity of >95%. The lyophilized peptide was stored at −20 °C. For identity confirmation via LC-MS analysis (200 ng total injection), a stock solution (0.5 mg/mL) was prepared in water containing a reducing agent (either 1 mM DTT or 500 µM TCEP). To analyze the oligomerization state of the peptide, a 1 mg/mL stock solution in water was diluted in either 50 mM phosphate buffer or 50 mM carbonate buffer (pH 7.4) to a final concentration of 25 µM before LC-MS injection (200 ng total). For cell-based assays, stock solutions (1 mg/mL) were prepared in water immediately before use, kept on ice during handling, and used within 24 h to prevent degradation or freeze–thaw related variability.

### 4.2. Cell Culture Conditions

The Human bronchial epithelial (BEAS-2B CRL-3588) cells were purchased from the American Type Culture Collection (ATCC, Manassas, VA, USA) and grown in DMEM/F12 (Gibco BRL, Grand Island, NY, USA) supplemented with 100× antibiotic solution (100 IU/mL penicillin and 100 μg/mL streptomycin; Himedia, Naples, Italy) and 10% Fetal Bovine Serum (Thermo Fisher Scientific, Waltham, MA, USA) and maintained at 37 °C with 5% CO_2_ in a humid environment.

### 4.3. Cytotoxic Activity

The cytotoxic profile of Ranalexin-1G was evaluated using 3-(4,5-dimethylthiazol-2-yl)-2,5-diphenyltetrazolium bromide (MTT) assay. BEAS-2B cells were seeded at a density of 2 × 10^4^ cells per well in 96-well plates and incubated for 24 h. The following day, the cell monolayer was treated with different peptide concentrations from 1.56 to 100 μM for 24 h. Untreated cells represented the negative control (CTR−), while cells treated with 100% DMSO represented the positive control (CTR+). The following day, a solution of MTT (5 mg/mL) was added to each well, and the plate was incubated for 3 h at 37 °C in a humidified atmosphere with 5% CO_2_. The solution was removed from each well, and 100 µL of 100% Dimethyl Sulfoxide (DMSO) was added to solubilize the formazan crystals [56]. Absorbance was recorded at 570 nm using a microplate reader (Tecan, Männedorf, Switzerland), and cell viability was calculated with the formula (Equation (1)):

(1) $$\%\;of\;cell\;viability=\;\frac{absorbance\;of\;treated\;cells}{absorbance\;of\;untreated\;cells}\;\times \;100$$

### 4.4. Hemolysis Assay

To further investigate the cytotoxic effects on human cells, the hemolytic activity of Ranalexin-1G was evaluated using fresh human erythrocytes obtained from healthy anonymous donors [57]. Briefly, 25 mL of whole blood was centrifuged, and the erythrocytes were washed three times with a 150 mM NaCl solution. The cells were then diluted 1:50 in phosphate-buffered saline (PBS, Sigma-Aldrich; pH 7.4), and 190 µL of the erythrocyte suspension was dispensed into each well of a 96-well conical-bottom plate. Ranalexin-1G was assessed at concentrations between 1.56 and 100 µM. Distilled water and 1% Triton X-100 solution were used as negative (CTR−) and positive (CTR+) controls, respectively. The samples were incubated at 37 °C under orbital shaking for 1 h and then centrifuged at 500 rpm for 5 min. Hemoglobin release was quantified by measuring the optical density of the supernatant at 540 nm, and the hemolysis percentage was calculated using the formula (Equation (2)):

(2) $$\%\;Hemolysis=\frac{absorbance\;of\;sample-absorbance\;of\;CTR-}{absorbance\;of\;CTR+\;-absorbance\;of\;CTR-}\;\times \;100$$

### 4.5. Bacterial Strains

The bacterial strains used in the current work were *P. aeruginosa* reference strain (*P. aeruginosa* ATCC 13388) and clinical isolates (CIs) of *P. aeruginosa* collected from cystic fibrosis patients, kindly provided by the Clinical Pathology Unit, S. Elia Hospital of Caltanisetta. Clinical isolates were identified using Matrix-Assisted Laser Desorption/Ionization–Time of Flight (MALDI-TOF, Bruker Daltonics, Bremen, Germany), and susceptibility tests were performed using the Phoenix BD system (Becton Dickinson, Franklin Lakes, NJ, USA). The features of the bacterial strains used in this study are listed in Table 2. The three clinical isolates exhibited distinct antimicrobial susceptibility profiles, reflecting different resistance phenotypes commonly observed in *P. aeruginosa* infections in CF patients. Clinical isolate 1 (CI1) was identified as a carbapenemase-producing strain (CARB). Carbapenemases are β-lactamase enzymes that hydrolyze carbapenems, which are considered last-resort antibiotics for the treatment of severe infections caused by Gram-negative bacteria. CI1 was resistant to aztreonam, cefepime, imipenem, and piperacillin, and showed intermediate susceptibility to levofloxacin and piperacillin–tazobactam. Notably, despite its carbapenemase-producing phenotype, CI1 remained susceptible to Meropenem. Clinical isolates 2 (CI2) and 3 (CI3) exhibited highly similar antimicrobial profiles, characterized by intermediate susceptibility to multiple β-lactams, including aztreonam, cefepime, ceftazidime, imipenem, piperacillin, and piperacillin–tazobactam, as well as to fluoroquinolones (ciprofloxacin and levofloxacin). In contrast to CI1, neither CI2 nor CI3 was classified as carbapenemase-producing.

### 4.6. Determination of the Minimum Inhibitory Concentration (MIC) and Minimal Bactericidal Concentration (MBC)

The antimicrobial activity of Ranalexin-1G was evaluated against both reference and clinical strains of *P. aeruginosa* using a microdilution assay. A single colony grown on Mueller-Hinton (MH) agar was inoculated into MH broth and incubated at 37 °C under shaking conditions (180 rpm) until reaching the mid-logarithmic phase. In the 96-well plate, the standardized bacterial inoculum (5 × 10^5^ CFU/mL) was exposed to different concentrations of peptide (from 25 to 1.56 μM) and incubated at 37 °C for 20 h. Meropenem (10 μg/mL = 25 μM) was used as a positive control (CTR+), while untreated bacteria constituted the negative control (CTR−) [58]. After incubation, optical density (OD) was measured using a Tecan reader, and the results were expressed as a percentage of growth inhibition, calculated via the following formula (Equation (3)):

(3) $$\%\;Growth\;Inhibition=1-\;\frac{Abs600\;of\;the\;test\;sample}{Abs600\;of\;CTR-}\;\times \;100$$

The minimum inhibitory concentration (MIC) was defined as the lowest peptide concentration that inhibited 95% of bacterial growth. After setting the MIC value, to assess bactericidal activity, 50 μL from the wells containing 1 × MIC and ½ × MIC were plated on MH agar and incubated at 37 °C for 18 h. The minimum bactericidal concentration (MBC) was defined as the lowest concentration able to kill 99.9% of the bacteria (detection limit ~20 CFU/mL).

### 4.7. Time-Killing Assay

To better understand the Ranalexin-1G kinetics of action against *P. aeruginosa*, the time-killing test was performed using the same conditions as the MIC assay. The effect of the peptide was observed at defined time intervals (0, 1, 2, 3, 6, and 18 h). Briefly, at each set time, 10 μL were taken from the wells (1 × MIC and ½ × MIC), and 10-fold serial dilutions in PBS were performed [59]. The different dilutions were plated on MH and incubated at 37 °C overnight (O/N). After incubation, the colonies were counted to define CFU/mL.

### 4.8. Analysis of Alginate Biosynthesis Gene Expression

To assess the expression of genes involved in alginate biosynthesis, the MIC test was performed as described above. The treatment was blocked after 24 h, and total RNA was extracted with TRIzol^®^ reagent (Thermo Fisher, Waltham, MA, USA) and quantified with Nanodrop spectrophotometer, with RNA quality assessed by A260/A280 ratio. Ana mount of 1 μg of total cellular RNA was reverse transcribed into complementary DNA (cDNA) using the 5× all-in-One RT mastermix (Applied Biological Materials, Richmond, BC, Canada). Real-Time PCR was performed in duplicate using the Insta Q96 (−6.0) thermocycler. Briefly, 2 μL of cDNA were amplified in a 20 μL reaction using BlasTaq 2 qPCR mastermix (Applied Biological Materials, Richmond, BC, Canada) and 0.1 μM of primers [60]. Primer efficiency was previously validated and found to be within acceptable limits. The mRNA levels of bacterial cells treated with the peptide were compared with those of untreated cells (control) and normalized with the internal housekeeping gene 16S. The 2-ΔΔCT method was employed to determine the threshold cycle (Ct) difference between the target and housekeeping genes. These genes were selected as representative markers of key steps in alginate biosynthesis and maturation. Specifically, *algA* and *algD* are involved in the early precursor synthesis pathway, *alg8* encodes a polymerase required for alginate polymerization, and *algG* is responsible for the modification of the polymer structure, thereby contributing to biofilm matrix maturation. Primers were purchased by Eurofins (Vimodrone, Milan, Italy) and their sequences are reported in Table 3.

### 4.9. Antibiofilm Assays

The antibiofilm activity of Ranalexin-1G was tested using two different assays to assess the peptide’s ability to inhibit biofilm formation and promote biofilm degradation.

#### 4.9.1. Biofilm Inhibition

A single colony of *P. aeruginosa* was inoculated into 5 mL of Luria–Bertani (LB) broth supplemented with 2% glucose and incubated for 20 h at 37 °C. The resulting culture was adjusted to an OD_600_ of 0.1, exposed to peptide concentrations ranging from 3.125 to 25 μM in a 96-well plate, and incubated for 20 h at 37 °C. Untreated bacteria represented the negative control (CTR−). After treatment, the wells were washed twice with PBS to remove non-adherent cells. Inhibition of biofilm formation was evaluated using the crystal violet (CV) assay. Specifically, 100 μL of 0.05% CV solution was added to each well and incubated at room temperature for 40 min. The wells were then rinsed with sterile water to remove excess dye, and 100 μL of ethanol (EtOH) was added to solubilize the bound stain for an additional 40 min. Finally, absorbance was measured at 570 nm using a Tecan microplate reader [61]. The percentage of inhibition was calculated using the following formula:

(4) $$\%\;Biofilm\;Inhibition=\;1-\;\frac{Abs570\;of\;the\;test\;sample}{Abs570\;of\;CTR-}\;\times \;100$$

#### 4.9.2. Biofilm Degradation

*P. aeruginosa* mature biofilms were established by inoculating 200 µL of a bacterial suspension adjusted to 10^8^ CFU/mL (OD_600_ = 0.2) into each well of a 96-well plate, followed by incubation for 20 h at 37 °C. The culture medium was replaced with a fresh medium containing different concentrations of peptides and incubated for another 20 h at 37 °C. Biofilm eradication activity was quantified using the CV colorimetric assay as described in Section 4.9.1 [62]. The percentage of biofilm degradation was calculated using the following formula:

(5) $$\%\;Biofilm\;Degradation=\;1-\;\frac{Abs570\;of\;the\;test\;sample}{Abs570\;of\;CTR-}\;\times \;100$$

### 4.10. Invasion Assay on Human Lung Epithelial Cells

BEAS-2B were seeded in 96-well plates. In the co-treatment assay, the cells were treated with Ranalexin-1G (12.5–6.25 µM) and simultaneously infected with *P. aeruginosa* ATCC 13388 at a MOI of 25 for 3 h. In pre-treatment, the cells were treated with Ranalexin-1G (12.5–6.25 µM) and incubated for 24 h. Subsequently, cells were infected with *P. aeruginosa* ATCC 13388 at an MOI of 25 for 3 h. In both experimental approaches, following infection, cells were washed with PBS1X and treated with DMEM/F12 medium containing 200 µg/mL of gentamicin sulfate (Sigma-Aldrich) for 1 h to eliminate extracellular bacteria. After antibiotic treatment, the cells were lysed with 0.1% Triton X-100 (Sigma-Aldrich) for 10 min at room temperature to count internalized bacteria. Serial 10-fold dilutions were prepared, spotted onto agar plates, and incubated for 18 h at 37 °C to determine the number of intracellular bacteria, expressed as colony-forming units (CFUs). Cells infected with *P. aeruginosa* (MOI = 25) were used as the negative control (CTR−), whereas infected cells treated with Meropenem (25 μM) served as the positive control (CTR+) [63].

### 4.11. Quantification of Bacterial Virulence Factors and Airway Mucin Gene

BEAS-2B cells were seeded in 96-well plates, treated with Ranalexin-1G (12.5–6.25 µM), and simultaneously infected with the *P. aeruginosa* reference strain at a MOI of 25 for 1 and 3 h. Supernatants collected after 3 h of infection were used for total bacterial RNA extraction using TRIzol^®^ reagent. The expression levels of the virulence genes *exoA*, *exoS*, and *lasA* (Table 4) were evaluated by real-time PCR as described in Section 4.8. In parallel, total RNA was also extracted from BEAS-2B cells to evaluate the gene expression of airway mucins *MUC1*, *MUC16*, and *MUC5AC* (Table 5). Cells infected with *P. aeruginosa* (MOI = 25) were used as a negative control (CTR−), while cells treated with Meropenem (25 µM) served as a positive control (CTR+). The mRNA levels of infected and treated cells were compared with those of untreated cells and normalized to the housekeeping gene GAPDH.

### 4.12. Scanning Electron Microscopy

Morphological studies of the bacterial surface were performed by SEM analysis. *P. aeruginosa* ATCC was exposed to Ranalexin-1G at the concentrations of 25–12.5 μM. After 6 h, the treatment was stopped, and the bacterial cells were recovered and centrifuged at 500 rpm for 15 min. Cell suspensions were fixed in 4% paraformaldehyde in phosphate buffer and dehydrated with increasing concentrations of ethanol (25, 50, 70, 95, and 100% *v*/*v*) [64].

### 4.13. LIVE/DEAD Fluorescence Staining

Bacterial membrane integrity was assessed using the LIVE/DEAD^®^ BacLight Bacterial Viability Kits (Invitrogen, Waltham, MA, USA), which contains the fluorescent nucleic acid stains SYTO™ 9 (3.34 mM) and propidium iodide (PI, 20 mM). Owing to their different membrane permeability properties, SYTO™ 9 stains all bacterial cells green, whereas PI penetrates only bacteria with compromised membranes, staining them red. *P. aeruginosa* was grown in MH broth and exposed to Ranalexin-1G at concentrations of 12.5 or 25 µM for 6 h. Untreated bacteria served as the negative control (CTR−). Following treatment, bacterial cultures were harvested by centrifugation at 10,000× *g* for 15 min, washed once, and finally resuspended in PBS1X. Immediately before staining, equal volumes of SYTO™ 9 and PI were mixed according to the manufacturer’s instructions. The dye mixture was added to the bacterial suspension at a final ratio of 3 µL per 1 mL of sample, followed by incubation for 15 min at room temperature in the dark [65]. Subsequently, 5 µL of the stained suspension was placed on a microscope slide and covered with an 18 × 18 mm coverslip. Fluorescence images were acquired using a ZOE™ Fluorescent Cell Imager (Bio-Rad Laboratories, Hercules, CA, USA).

### 4.14. Salt Resistance Assay

The clinical application of AMPs is often limited by the inhibitory effect of cationic salts on their antimicrobial activity, a phenomenon known as salt sensitivity. To assess this effect, the activity of Ranalexin-1G against a reference strain and clinical isolates of *P. aeruginosa* was evaluated using a broth microdilution assay in the presence of 150 and 300 mM NaCl, as previously described in Section 4.6 [66,67].

### 4.15. Statistical Analysis

All experiments were conducted in triplicate and reported as mean ± standard deviation (SD) calculated with GraphPad Prism (version 8.0.1). Statistical analysis was performed using One-way ANOVA followed by Dunnett’s multiple comparisons test. *p*-values < 0.001 were considered significant.

## 5. Conclusions

In summary, Ranalexin-1G is a multifunctional agent that combines direct bactericidal activity with potent anti-virulence and anti-invasive properties. Its ability to disrupt alginate biosynthesis and suppress host mucin overproduction makes it a promising candidate for treating *P. aeruginosa* respiratory infections, where traditional antibiotics often fail due to biofilm formation and host-mediated tissue damage.

## Figures and Tables

**Table 1:** Minimal inhibitory and bactericidal concentration (MIC and MBC, respectively) of Ranalexin-1G against *P. aeruginosa* ATCC 13388 and three clinical isolates (CI1-3).

Ranalexin-1G	MIC (μM)	MIC (μg/mL)	IC_50_ (μM)	MBC (μM)
*P. aeruginosa* ATCC 13388	25	59.85	11	25
*P. aeruginosa* Clinical isolate 1 (CI1)	12.5	29.93	4.11	25
*P. aeruginosa* Clinical isolate 2 (CI2)	25	59.85	20.9	-
*P. aeruginosa* Clinical isolate 3 (CI3)	25	59.85	12	-

**Table 2:** Susceptibility profile of *P. aeruginosa* collected clinical strains. AK: Amikacin; ATM: Aztreonam; CPM: Cefepime; FDC: Cefiderocol; CAZ: Ceftazidime; CZA: Ceftazidime-avibactam; CIP: Ciprofloxacin; CS: colistin; IMP: Imipenem; LVX: Levofloxacin; MEM: Meropenem; PRL: Piperacillin; TZP: Piperacillin-Tazobactam; TM: Tobramycin. S: Susceptible, I: Intermediate, R: Resistant.

Bacterial Strain	Resistance Profile	Clinical Specimen
*P. aeruginosa* ATCC 13388	Standard strain	-
*P. aeruginosa* clinical isolate 1 (CI1) CARB (carbapenemase-producing strain)	AK (S), ATM (R), CPM (R), FDC (S), CAZ (S), CZA (S), CIP (S), CS (S), IMP (R), LVX (I), MEM (S), PRL (R), TZP (I), TM (S)	Sputum
*P. aeruginosa* clinical isolate 2 (CI2)	AK (S), ATM (I), CPM (I), FDC (S), CAZ (I), CZA (S), CIP (I), CS (S), IMP (I), LVX (I), MEM (S), PRL (I), TZP (I), TM (S)	Sputum
*P. aeruginosa* clinical isolate 3 (CI3)	AK (S), ATM (I), CPM (I), FDC (S), CAZ (I), CZA (S), CIP (I), CS (S), IMP (I), LVX (I), MEM (S), PRL (I), TZP (I), TM (S)	Sputum

**Table 3:** Sequences of the alginate primers used for the real-time PCR.

Genes	Forward Sequence (5′–3′)	Reverse Sequence (5′–3′)
*algD*	CGTCTGCCGCGAGATCGGCT	GACCTCGACGGTCTTGCGGA
*alg8*	AGAGCTTCATCAAGGCCAGT	GGAAGGCGATGCTGTACTTG
*algG*	TTCCGTCCGTTCCTGATCTC	GCAATAGAAGCCGTACCAGC
*algA*	AGTAATCCTTTCCGGTGGCA	AGCTGTTCCTGGACGATGAA
*16S*	ATTTGAAGAGGTTTGCAAACGAT	TTCACTCTGAAGTTTCTTGTGTTC

**Table 4:** Sequences of the virulence factor primers used for the real-time PCR.

Genes	Forward Sequence (5′–3′)	Reverse Sequence (5′–3′)
*exoA*	GACAACGCCCTCAGCATCACCAGC	CGCTGGCCCATTCGCTCCAGCGCT
*exoS*	CTACACCGGCATTCACTAC	AAGTCTTCACTACCTGTTCAG
*lasA*	GCAGCACAAAAGATCCC	GAAATGCAGGTGCGGTC

**Table 5:** Sequences of airway mucin primers used for the real-time PCR.

Genes	Forward Sequence (5′–3′)	Reverse Sequence (5′–3′)
*MUC1*	CCTTTCTTCCTGCTGCTG	GGGCACTGAACTTCTCTG
*MUC16*	GATGTCAAGCCAGGCAGCACAA	GCCCAGAAATGTCTACCACTCTC
*MUC5AC*	TCCGGCCTCATCTTCTCC	CAGCACCAGTGCCCAAGT
*GAPDH*	CGGAGTCAACGGATTTGGTCGTAT	AGCCTTCTCCATGGTGGTGAAGAC

## Data Availability

The original contributions presented in this study are included in the article/Supplementary Material. Further inquiries can be directed to the corresponding author.

## Supplementary Materials

The following supporting information can be downloaded at: https://www.mdpi.com/article/10.3390/antibiotics15070711/s1, Figure S1: Evaluation of the peptide redox state over time.

## Abbreviations

The following abbreviations are used in this manuscript:

AAMPs	Anionic Antimicrobial Peptides
ABC	ATP-Binding Cassette
ATM	Aztreonam
CAZ	Ceftazidime
CARB	Carbapenemase-Producing Strain
CAMPs	Cationic Antimicrobial Peptides
CF	Cystic Fibrosis
CFTR	Cystic Fibrosis Transmembrane Conductance Regulator
CFU	Colony Forming Unit
CI	Clinical Isolate
CIP	Ciprofloxacin
cDNA	Complementary DNA
CPM	Cefepime
CV	Crystal violet
CZA	Colistin
DCM	Dichloromethane
DIC	Diisopropylcarbodiimide
DMEM/F12	Dulbecco’s Modified Eagle Medium/Nutrient Mixture F-12
DMF	Dimethylformamide
DMSO	Dimethyl Sulfoxide
EF-2	Elongation factor 2
EPS	Extracellular Polymeric Substances
ExoA	Exotoxin A
ExoS	Exoenzyme S
FDC	Cefiderocol
Fmoc	Fluorenylmethyloxycarbonyl
HOBt	Hydroxybenzotriazole
HPLC	High-Performance Liquid Chromatography
IMP	Imipenem
LB	Luria–Bertani
LC-MS	Liquid Chromatography–Mass Spectrometry
LasA	Elastase A
LVX	Levofloxacin
MBC	Minimum Bactericidal Concentration
MDR	Multidrug-Resistant
MEM	Meropenem
MH	Mueller-Hinton
MIC	Minimum Inhibitory Concentration
MOI	Multiplicity of Infection
MTT	3-(4,5-dimethylthiazol-2-yl)-2,5-diphenyltetrazolium bromide
MUC	Mucin
NE	Neutrophil elastase
OD	Optical density
Oxyma	Optical density
PBS	Phosphate-Buffered Saline
PI	Propidium iodide
PRL	Piperacillin
RP-HPLC	Reverse-Phase High-Performance Liquid Chromatography
RT-PCR	Reverse Transcription Polymerase Chain Reaction
SD	Standard Deviation
SEM	Scanning Electron Microscopy
TSS	Type Secretion System
TFA	Trifluoroacetic Acid
THF	Tetrahydrofuran
TIS	Triisopropylsilane
TM	Tobramycin
TZP	Piperacillin-Tazobactam
