# Supplementary material for: Ranalexin-1G: A Promising Antimicrobial Peptide Targeting Virulence and Host–Pathogen Interactions in Pseudomonas aeruginosa In Vitro Models
Source: Antibiotics (Basel). 2026 Jul 22;15(7):711. doi: 10.3390/antibiotics15070711 (PMC13403631; doi:10.3390/antibiotics15070711)
Supplement: Supplementary file 1 [file antibiotics-15-00711-s001.zip › antibiotics-4422932-supplementary.pdf]

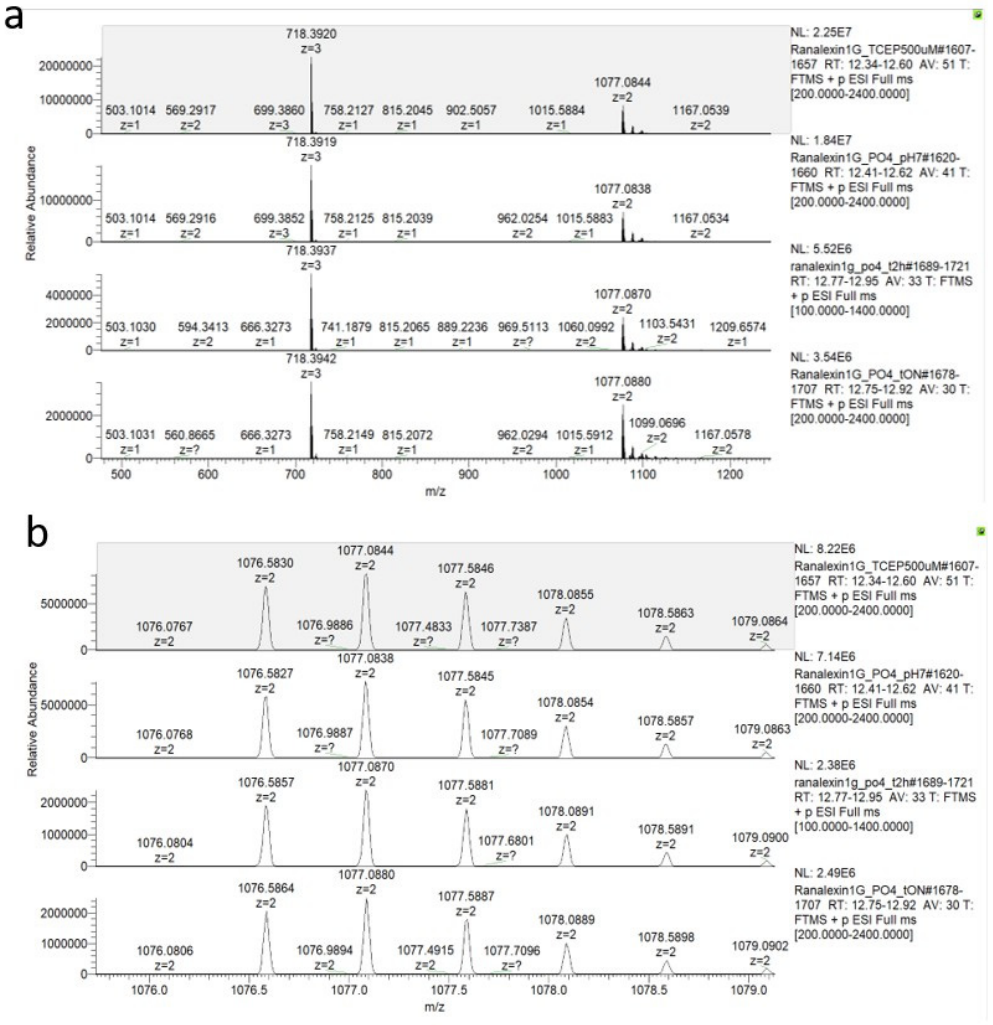

Supplementary Figure S1. Evaluation of the peptide redox state over time. (a) Top: m/z spectrum of the peptide in 500 mM TCEP in phosphate buffer (reference). Bottom: mass spectra of the peptide without reducing agent at 0 h, 2 h, and 24 h. (b) Expanded view of the isotopic profile for the peak at m/z 1077.08 from each spectrum shown in panel (a), confirming the reduced state of the peptide; panels are arranged in the same sequential order. Similar results were observed in carbonate buffer at pH 7.4 (data not shown).
